# Supplementary material for: Aerosolized Exposure to H5N1 Influenza Virus Causes Less Severe Disease Than Infection via Combined Intrabronchial, Oral, and Nasal Inoculation in Cynomolgus Macaques
Source: Viruses. 2021 Feb 22;13(2):345. doi: 10.3390/v13020345 (PMC7926951; doi:10.3390/v13020345)
Supplement: Supplementary file 1 [file viruses-13-00345-s001.pdf]

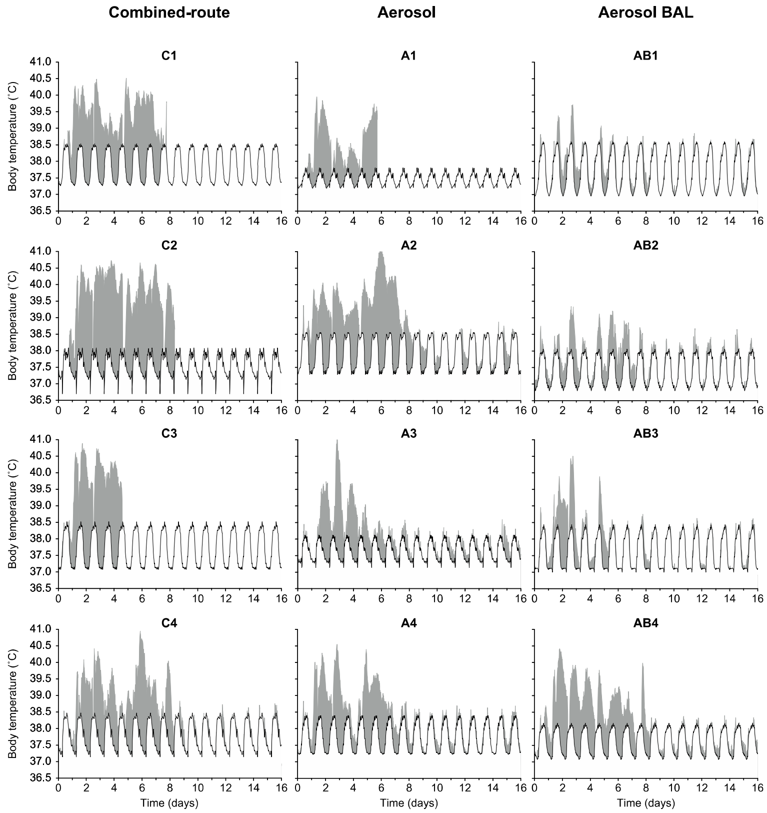

Supplemental Figure 1. Body temperature of individual animals. Shown in grey is the actually recorded temperature in time after infection for each individual animal. These temperatures are plotted on top of the circadian temperature pattern recorded before infection (white area under the black line). The circadian pattern was calculated from the temperatures recorded during seven days before infection and plots show the calculated mean temperature plus the upper 95% confidence interval
